# Fidelity to Program Specification of the National Health Service Digital Diabetes Prevention Program Behavior Change Technique Content and Underpinning Theory: Document Analysis

**DOI:** 10.2196/34253

**Published:** 2022-04-27

**Authors:** Rhiannon E Hawkes, Lisa M Miles, David P French

**Affiliations:** 1 Manchester Centre for Health Psychology Division of Psychology and Mental Health University of Manchester Manchester United Kingdom

**Keywords:** diabetes prevention, digital interventions, behavior change, fidelity

## Abstract

**Background:**

The National Health Service (NHS) Diabetes Prevention Program is a behavior change intervention for adults in England who are identified as being at high risk of developing type 2 diabetes. The face-to-face service was launched in 2016, followed by a digital service (NHS Digital Diabetes Prevention Program [NHS-DDPP]) in 2019. A total of 4 service providers were commissioned to deliver the NHS-DDPP and were required to deliver the digital service in line with a program specification detailing the key intervention content. The fidelity of the behavior change content in the digital service (ie, the extent to which the program is delivered as intended) is currently unknown. Digital interventions may allow higher fidelity as staff do not have to be trained to deliver *all* intervention content. Assessing fidelity of the intervention design is particularly important to establish the planned behavior change content in the NHS-DDPP and the extent to which this adheres to the program specification. This is the first known independent assessment of design fidelity in a large-scale digital behavior change intervention.

**Objective:**

This study aims to assess the fidelity of the behavior change content in each of the 4 NHS-DDPP providers’ intervention designs to the full program specification.

**Methods:**

We conducted a document review of each provider’s NHS-DDPP intervention design, along with interviews with program developers employed by the 4 digital providers (n=6). Providers’ intervention design documents and interview transcripts were coded for behavior change techniques (BCTs; ie, the *active ingredients* of the intervention) using the Behavior Change Technique Taxonomy version 1 and underpinning theory using the Theory Coding Scheme framework. The BCTs identified in each digital provider’s intervention design were compared with the 19 BCTs included in the program specification.

**Results:**

Of the 19 BCTs specified in the program specification, the 4 providers planned to deliver 16 (84%), 17 (89%), 16 (84%), and 16 (84%) BCTs, respectively. An additional 41 unspecified BCTs were included in at least one of the 4 digital providers’ intervention designs. By contrast, inconsistent use of the underpinning theory was apparent across providers, and none of the providers had produced a logic model to explain how their programs were expected to work. All providers linked some of their planned BCTs to theoretical constructs; however, justification for the inclusion of other BCTs was not described.

**Conclusions:**

The fidelity of BCT content in the NHS-DDPP was higher than that previously documented for the face-to-face service. Thus, if service users engage with the NHS-DDPP, this should increase the effectiveness of the program. However, given that a clear theoretical underpinning supports the translation of BCTs in intervention designs to intervention delivery, the absence of a logic model describing the constructs to be targeted by specific BCTs is potentially problematic.

## Introduction

### Background

Type 2 diabetes mellitus (T2DM) is an international public health concern, with >4.9 million people having diabetes and 13.6 million people now at an increased risk of T2DM in the United Kingdom [1]. T2DM is preventable by lifestyle modifications in diet, physical activity, and weight loss, although family history, ethnicity, and age are risk factors. Treatment for T2DM and its associated complications currently costs the National Health Service (NHS) £10 billion (US $13 billion) each year; thus, its prevention is a public health priority [2].

In response to this, the NHS Diabetes Prevention Program (NHS-DPP) was launched in 2016, a behavior change intervention for adults in England who have been identified as having elevated blood glucose levels and are thus at a higher risk of developing T2DM. The main aims of the program are to improve diet, increase physical activity, and achieve weight loss, contributing to a reduction in T2DM risk [3]. Early evaluations suggest that the program is successful in preventing the progression to T2DM [4]. However, there is a weakness in reach and retention in the face-to-face program (eg, younger adults and those from more deprived communities) [5], which a digital program may overcome. Digital interventions have the potential to address logistical challenges such as scheduling, travel, work, and childcare [6], and they have the capacity to offer wide-reaching and tailored support at a lower cost, both of which are important for large-scale behavior change [7]. For example, technology can offer more choice and convenience to patients who may not be able to engage face to face with health professionals, and interventions can be designed to address the specific needs of disadvantaged groups by tailoring those interventions to the target group [8], especially when these groups are included in the design of digital interventions [9].

In 2017, NHS England launched a pilot program of the NHS Digital DPP (NHS-DDPP) [10]. Following this pilot, a digital pathway was introduced to the program in 2019 [11], with 4 independent service providers commissioned to deliver the digital service. NHS England produced a service specification detailing the key features that should be present in the NHS-DPP [11], based on the currently available evidence [12,13]. This specification indicated the key behavior change techniques (BCTs) that should be present in the intervention [11]. BCTs are defined as the *active ingredients* of an intervention that promotes behavioral change (eg, setting goals, monitoring behavior, and social support) [14]. The use of BCTs to self-regulate behaviors (eg, goal setting and self-monitoring) was particularly emphasized in the specification, as the wider literature suggests that these are key to behavioral change [13].

Intervention fidelity is used to describe whether an intervention is *delivered as intended* [15,16]. Without an assessment of fidelity, it cannot be ascertained whether intervention effectiveness, or a lack thereof, is because of the intervention content or other factors added or omitted in the intervention delivery. A recent evaluation of the face-to-face delivery of the NHS-DPP produced a thorough fidelity assessment of the behavior change content in the program, including the assessment of intervention design [17], staff training [18], intervention delivery [19-21], and service user receipt [22]. The results of the face-to-face evaluation revealed an underdelivery of BCTs to self-regulate behaviors (eg, problem solving and goal setting) [19,21]. This suggests a gap between what the evidence base indicated as the most effective in changing health behaviors and actual program delivery, which is largely because of failures to translate the BCTs specified in the evidence base into providers’ program designs [19].

An assessment of the fidelity of the design of interventions is an important first stage in fidelity evaluation to establish what key intervention features are planned and whether these are in line with the evidence base from the outset. If a program is not designed with fidelity to the evidence base, it will have a downstream influence on its delivery and receipt. The findings from the assessment of fidelity of design for the face-to-face service indicated 19 BCTs in the NHS-DPP full program specification that should be included in the program; face-to-face providers described 74% of those BCTs in their intervention designs [17]. However, we are yet to establish the fidelity of the behavior change content in the digital offering of the NHS-DPP; that is, the extent to which the digital providers include the 19 specified BCTs in their intervention designs.

The underpinning theory of an intervention refers to the theories that inform the intervention, such as identifying theoretical constructs to be targeted [23]. Having identified these constructs allows for appropriate BCTs to be selected. Despite recommendations for clear descriptions of the underpinning theory of behavior change interventions [24], there is still a lack of literature that reports how interventions work (ie, the theoretical basis of the intervention and the rationale for selecting the BCTs designed for the intervention). To facilitate the description of different aspects of theory use, the Theory Coding Scheme (TCS) was developed [25], which is a 19-item framework providing a fine-grained assessment of the use of theory, including the extent to which all mentioned BCTs are linked to relevant constructs.

A concise way of visually presenting the theoretical basis for interventions that are informed by multiple theories could be via a logic model that uses simple diagrams to demonstrate the causal pathways between the intervention components and desired outcomes [26]. Without an explicit underpinning theory, it is not clear how the program is expected to produce the desired behavioral changes; thus, the rationale for the design of the intervention is not clear, and there is a lack of justification for why particular BCTs have been chosen for the intervention [25]. The evaluation of the face-to-face NHS-DPP found variation in the underpinning theory across face-to-face providers, with a lack of justification for the inclusion of BCTs in providers’ intervention designs [27].

Digital interventions may allow higher fidelity as staff do not have to be trained to deliver *all* the intervention content [7]. To date, research has primarily focused on patient engagement with digital health interventions (eg, via methods such as system usage data, qualitative measures, and self-report questionnaires) [28] and adherence to digital interventions (ie, the extent to which patients keep using the digital intervention in the desired way) [29]. Some studies have summarized the development process of digital interventions (eg, describing the theory and existing evidence to inform the development of a digital self-management intervention for people with T2DM) [30] and compared web-based intervention content against the intervention description [31]. However, there is a lack of literature examining the extent to which the planned intervention content of digital health interventions adheres to the evidence base for what works in changing health behaviors.

### Objectives

Assessing the fidelity of nationally implemented digital behavior change interventions is rare, and to the best of our knowledge, none to date have assessed the fidelity of design to behavior change content. This analysis aims to provide the first known independent evaluation of fidelity of design of a nationally implemented digital intervention and thus builds on previous methods set out to measure fidelity [16] by applying them in a novel way to digital intervention designs using the NHS-DDPP as an exemplar. Therefore, the objectives of this study are to (1) describe the planned BCT content and theoretical foundations of the NHS-DDPP, (2) examine variations in planned BCT content and theoretical foundations between the digital providers, and (3) evaluate the fidelity of the planned BCT content in each provider’s NHS-DDPP intervention design to the full program specification.

## Methods

### Design

We conducted a document analysis of the NHS-DDPP providers’ intervention design documentation, along with qualitative interviews with program developers of each of the 4 digital providers. There were no single documents for any digital provider that offered a full and complete summary of their intervention designs; thus, interviews were used to supplement the documentation to allow researchers to gain in-depth information about the development of the NHS-DDPP and the implementation journey of the digital program. Digital providers’ intervention designs were compared with the NHS-DPP program specification.

### Document Review

#### Program Specification Documentation

The program specification documentation indicated the key intervention content that should be included in the NHS-DDPP. The documents that comprised the program specification are described in the following sections.

##### NHS-DPP Service Specification

This document [11] was specific to the commissioning of the NHS-DPP and was based on an evidence review of lifestyle interventions for the prevention of type 2 diabetes [12]. It specified what NHS England required to be included in the NHS-DPP (including face-to-face and digital offerings), drawing on recommendations from the National Institute for Health and Care Excellence (NICE) PH38 guidance [13].

##### The NICE PH38 Guideline, Type 2 Diabetes: Prevention in People at High Risk

This document [13] provided additional information regarding behavior change content to be included in diabetes prevention programs and was referred to in the NHS Service Specification [11].

The research team also consulted the recently published NICE NG183 guideline, *Behavior change: digital and mobile health interventions* [32], providing guidance on digital intervention development targeting health behaviors, including diet and physical activity [32]. However, there was no additional information regarding the specific behavior change content that was not already included in the NICE PH38 guidance [13]. Thus, the NG183 guideline [32] has not been included as a specification document in the current research. Multimedia Appendix 1 provides further justification.

#### Digital Providers’ Intervention Design Documentation

The following documents from digital providers described providers’ intervention designs obtained between June 2020 and February 2021:

- Providers’ framework response bids describing digital providers’ proposed service delivery submitted to NHS England during service procurement; 1 per digital provider (n=4)
- Additional documentation supplied by 75% (3/4) of digital providers, which detailed further information about the planned behavior change content designed in their digital programs (Table 1)
- Email correspondence with 25% (1/4) of digital providers containing further details of the BCTs and the underpinning theory of their digital programs; this was obtained when program developers were interviewed and sought further information from other colleagues who were not available to be interviewed but could provide further details to some of the interview questions via email (Table 1)

**Table 1:** Data obtained from each digital provider.

Digital provider	Number of interviews	Further information obtained from participant via email?	Further documentation obtained from provider?
A	2	Yes	Yes
B	1	No	Yes
C	2	No	No
D	1	No	Yes

### Participants

For the purpose of this manuscript, the term *digital providers* indicates the 4 commercial companies commissioned to deliver the NHS-DDPP. The 4 digital providers were private service organizations, each of whom secured contracts to deliver the NHS-DDPP from 2019 to 2021. Of the 4 digital providers, 3 (75%; Oviva, Second Nature, and Liva) were in partnership with one of the face-to-face providers of the NHS-DPP to deliver the digital pathway of the program. One of the providers (WW) delivered both face-to-face and digital pathways of the NHS-DPP. In this report, providers are labeled A to D to preserve their anonymity.

To supplement the digital providers’ intervention design documentation, interviews were conducted between September 2020 and December 2020 with program developers employed by each of the 4 digital providers. Program developers were involved in the design and development of the NHS-DDPP. Participants had job titles including Head of NHS Partnerships, Head of Clinical Services, and Head of Coaching. Participants were involved in either adapting their current digital programs to be in line with the NHS Service Specification [11] or were a key contact at the digital provider best placed to describe how the behavior change content was developed for the program.

### Ethics Approval

The wider program of research of which this study is a part of was reviewed and approved by the North West Greater Manchester East NHS Research Ethics Committee (reference 17/NW/0426; August 1, 2017).

### Procedures and Recruitment

The authors were in contact with the management staff of each of the 4 digital providers to obtain all relevant documentation detailing the providers’ intervention designs. All documents were emailed to the research team.

The authors contacted the management staff of each of the 4 digital providers to help identify the appropriate staff members to be interviewed. The management staff contacted the relevant individuals directly, and those individuals proactively contacted the research team, where they expressed an interest in participating in an interview. We aimed to interview professionals from different backgrounds who had different roles in program development (eg, specialists with backgrounds in behavioral science, nutrition, or physical activity, and provider leads overseeing program development) to gather a range of views and provide a comprehensive understanding of the processes involved in the design and development of each NHS-DDPP intervention. Before scheduling the interviews, the participants were sent an information sheet containing details about the research. Participants were given the opportunity to ask any questions about the research, and fully recorded verbal consent was obtained from each participant before commencing the interview. Interviews were conducted via a video call platform—Zoom (Zoom Video Communications) videoconferencing—with one of the two researchers (REH and LMM) who had previous training in qualitative data collection.

Interviews with 2 professionals took place with digital providers A and C, and interviews with 1 professional took place with digital providers B and D (n=6 interviews in total). The interviews covered the following topics:

- Participants’ professional backgrounds and roles in the development of the NHS-DDPP
- The theoretical underpinning of their programs, including targeted constructs and why specific theories or models were chosen
- Planned BCTs in the program, including behaviors targeted by these techniques and how these techniques were expected to work in producing behavioral changes
- The extent to which the programs were adapted from pre-existing digital programs for the NHS-DDPP
- The extent to which the digital offering of the NHS-DPP changed from the face-to-face offering of the program
- Strategies in place to support service users throughout the program (eg, at first contact and continued engagement)
- Content of the digital intervention and format of the different intervention features included in the program

The interviews were semistructured; the researchers initially asked open questions about the general topic, followed by a more detailed probing on specific issues. It was noted that participants were not expected to be able to answer all the interview questions, depending on their specific role and expertise in intervention development. Interviews were transcribed verbatim. Taken together, the documentation from each digital provider and interviews with program developers described the behavior change content that digital providers intended to deliver in each of their programs (providers’ intervention designs).

### Data Analysis

#### Coding Frameworks

A content analysis of BCTs and the underpinning theory contained within all documentation and interview transcripts was conducted using the following coding frameworks: the Behavior Change Technique Taxonomy version 1 (BCTTv1) [14] to document the BCTs described in providers’ digital intervention designs and the TCS [25] to document the underpinning theory and theoretical constructs described in providers’ digital intervention designs.

The BCTTv1 [14] lists 93 distinct techniques, each with its own labels and definitions, categorized into 16 groups. The TCS [25] is a 19-item framework that captures how theory is directly used in the intervention (eg, links between theoretical constructs and BCTs). Both frameworks have been widely used for reporting interventions; the BCTTv1 has demonstrated good interrater reliability (IRR), test-retest reliability, and good validity [33], and the authors of the TCS have reported satisfactory IRR for each item of the TCS [25].

The semistructured interviews were analyzed using deductive content analysis. Information relating to the behavior change content of the program, including BCTs and the underpinning theory, was extracted from all documentation and interview transcripts using these coding frameworks.

#### Coding Procedures

Providers’ intervention design documentation and interview transcripts were coded separately using the BCTTv1 [14] and TCS [25] by one researcher (REH). BCTs were coded using an author-developed data extraction sheet. Researchers underwent training in the use of the BCTTv1 [34], and a set of coding rules was developed through team discussions. BCTs present in the program specification documentation [11,13] were coded for a previous fidelity assessment of the face-to-face offering of the NHS-DPP conducted by the same research team [17]. The study by Hawkes et al [17], as well as Multimedia Appendix 2, provides further details on the developed BCT coding rules. To code the underpinning theory, a data extraction sheet was developed using the TCS [25]. Multimedia Appendix 2 provides further details on how the data on the underpinning theory were extracted.

The framework response bids from the digital providers’ intervention design documentation were double coded for BCTs and the underpinning theory by a second researcher (LMM). IRR was calculated using the Cohen κ coefficient [35] to determine consistency between coders. Identified coding discrepancies were discussed among REH, LMM, and DPF until consensus was reached. Cohen κ values were previously determined for the NHS Service Specification [11] and NICE PH38 guideline [13] (program specification documentation) by Hawkes et al [17]. Multimedia Appendix 3 provides all κ values.

#### Fidelity Analysis

The BCTs present in the NHS Service Specification and NICE guidance (program specification) were compared with those present in providers’ intervention design documentation and interview transcripts (digital providers’ intervention designs), and the results were tabulated. The theoretical principles detailed in digital providers’ intervention designs were summarized for each provider.

## Results

### BCT Content

#### Program Specification Documentation

A previous analysis of the NHS-DPP program specification indicated 19 BCTs that should be included in the NHS-DPP intervention [17] (Table 2). The program specification emphasized the use of self-regulatory techniques, including goal setting, action planning, problem solving, and self-monitoring.

**Table 2:** Behavior change techniques specified in the full program specification compared with behavior change techniques specified in digital providers’ intervention designs^a^.

Behavior change techniques^b^	NICE^c^ PH38	NHS^d^ Service Specification	A	B	C	D
Credible source	✓^e^	✓	✓	✓	✓	✓
Goal setting (behavior)	✓	✓	✓	✓	✓	✓
Goal setting (outcome)	✓	✓	✓	✓	✓	✓
Graded tasks	✓	✓	✓	✓	✓	✓
Information about health consequences	✓	✓	✓	✓	✓	✓
Social support (unspecified)	✓	✓	✓	✓	✓	✓
Action planning	✓		✓	✓	✓	✓
Behavior substitution	✓		✓	✓	✓	✓
Problem solving	✓		✓	✓	✓	✓
Review outcome goals	✓		✓	✓	✓	✓
Self-monitoring of behavior	✓		✓	✓	✓	✓
Self-monitoring of outcomes of behavior	✓		✓	✓	✓	✓
Social support (emotional)^f^	✓		✓	✓	✓	✓
Feedback on behavior	✓		✓	✓	✓	
Social support (practical)^f^	✓			✓	✓	✓
Behavioral practice or rehearsal		✓	✓	✓	✓	✓
Monitoring of outcomes of behavior without feedback		✓		✓		
Pros and cons	✓		✓			✓
Pharmacological support		✓				
Commitment			✓	✓^g^	✓	✓
Demonstration of behavior			✓	✓	✓	✓
Feedback on outcomes of behavior			✓	✓	✓	✓
Habit formation			✓	✓	✓	✓
Instruction on how to perform the behavior			✓	✓	✓	✓
Reduce negative emotions			✓	✓	✓	✓
Restructuring the physical environment			✓^h^	✓	✓	✓^h^
Review behavior goals			✓	✓	✓	✓
Reward (outcome)			✓^i^	✓^i^	✓^i^	✓
Social reward			✓^i^	✓	✓^i^	✓
Framing and reframing			✓	✓^g^	✓	
Identification of self as a role model			✓	✓	✓	
Restructuring the social environment			✓^h^	✓		✓^h^
Social comparison			✓	✓		✓
Biofeedback			✓		✓	✓
Information about antecedents			✓		✓	✓
Prompts and cues			✓		✓	✓
Behavioral contract				✓	✓	✓
Focus on past success				✓	✓	✓
Self-talk				✓^g^	✓	✓
Increase positive emotions^j^			✓	✓		
Verbal persuasion about capability				✓^g^	✓	
Information about emotional consequences				✓		✓
Avoidance or reducing exposure to cues for the behavior				✓		
Behavioral experiments				✓^g^		
Material incentive (behavior)				✓		
Material reward (behavior)				✓		
Mental rehearsal of a successful performance				✓		
Monitoring of emotional consequences				✓		
Nonspecific incentive				✓^g^		
Remove aversive stimulus				✓		
Reward alternative behavior				✓		
Self-incentive				✓		
Self-reward				✓^g^		
Information about social and environmental consequences					✓	
Salience of behaviors^k^					✓	
Vicarious consequences					✓	
Adding objects to the environment						✓
Body changes						✓
Habit reversal						✓
Social incentive						✓

^a^The data in this table combine results from design documentation supplied by providers and interviews with program developers involved in the design of the digital diabetes prevention program.

^b^The first 19 behavior change techniques (from *Credible source* to *Pharmacological support*) are those 19 core behavior change techniques specified in the full program specification underpinning the NHS Diabetes Prevention Program.

^c^NICE: National Institute for Health and Care Excellence.

^d^NHS: National Health Service.

^e^Addition from coding of updated guidance when the document was updated in September 2017.

^f^*Social support (practical)* and *Social support (emotional)* were coded as 1 behavior change technique in the NICE guideline, as it stated that either of these forms of social support could be delivered.

^g^*Framing and reframing*, *Self-talk*, *Verbal persuasion about capability*, *Nonspecific incentive*, *Self-reward*, *Commitment*, and *Behavioral experiments* were present in optional extra sessions only for this provider.

^h^*Restructuring the physical environment* and *Restructuring the social environment* were coded as 1 behavior change technique in the framework response, as it stated only to restructure the environment without specifying whether this was the physical or social environment.

^i^*Social reward* and *Reward (outcome)* were coded as 1 behavior change technique in the framework response, as it did not state whether the reward was for the behavior or outcome.

^j^Increased positive emotions are not listed in the Behavior Change Technique Taxonomy version 1 but were noted by the authors for inclusion in the next version of the taxonomy and used in Hawkes et al [17].

^k^Salience of behaviors was not listed in the Behavior Change Technique Taxonomy version 1 but has been identified as a new behavior change technique by the authors of this paper and used in Hawkes et al [17].

#### Digital Providers’ Intervention Designs

Cohen κ values ranged from 0.66 to 0.93 for the double coding of BCTs in digital providers’ intervention design documentation, demonstrating moderate to almost perfect agreement between coders [36] before resolving discrepancies (see Multimedia Appendix 3 for all κ values). Table 2 shows the BCTs identified in each of the 4 digital providers’ intervention designs based on all supplied documentation and interviews with the program developers of each digital provider.

Combining the content analysis of all documentation and interview data, providers A, B, C, and D planned to deliver 34, 48, 38, and 39 unique BCTs, respectively, in their digital programs. For provider B, 15% (7/48) of these BCTs were present only in optional extra sessions. All 4 providers planned to deliver 23 common BCTs, including action planning, goal setting (for behaviors and outcomes), graded tasks, information about health consequences, problem solving, reviewing goals (for behaviors and outcomes), self-monitoring (for behaviors and outcomes), and social support (emotional and unspecified). Multimedia Appendix 4 provides BCT definitions according to the BCTTv1 [14].

#### Fidelity of BCT Content

There were 19 BCTs specified in the program specification documentation [11,13]. Of these 19 BCTs, the 4 providers included 16 (84%), 17 (89%), 16 (84%), and 16 (84%) BCTs in their intervention designs, respectively. Thus, overall, the mean proportion of BCTs in digital providers’ intervention designs to the program specification was 85% (SD 2.5%). Of the 19 BCTs required by the full program specification, 14 (74%) were included in the intervention designs of all 4 digital providers (Table 2). These included self-regulatory techniques such as goal setting, action planning, problem solving, and self-monitoring.

A total of 6 BCTs specified in the program specification documentation were missing from at least one digital provider’s intervention designs, indicating a lack of fidelity to the program specification by BCT omission. One of the BCTs, pharmacological support (eg, advising on medication for smoking cessation), was not provided by any of the 4 digital providers. There were 41 additional BCTs not indicated in the program specification, which were included in at least one of the 4 digital providers’ intervention designs.

### Underpinning Theory

Across the 4 digital providers, the mean κ value for coding using the TCS was 0.90, and the mean κ values for theories and constructs mentioned were 1.00 and 0.97, respectively. Cohen κ values demonstrated strong to an almost perfect agreement [36] between coders using TCS before resolving discrepancies (Multimedia Appendix 3 provides all κ values).

The extent to which theory was used in each of the 4 providers’ intervention designs is summarized in Table 3. All digital providers mentioned theories on which their programs were based; however, none had produced an explicit logic model of how their program was expected to produce changes in behavior. Providers A, B, and D stated that their digital programs were informed by the Capability, Opportunity, Motivation–Behavior framework [37], with providers B and D describing a particular emphasis on this model. Providers A and C cited multiple theories to inform their intervention designs, including Social Cognitive Theory [38], the Transtheoretical Model [39], and Self-Determination Theory [40] (Table 4).

There was wide variation in the constructs that providers were targeting in their digital programs. All 4 digital providers mentioned the constructs of goals, motivations, and capabilities in their intervention designs. The following constructs were also mentioned in the digital designs of 75% (3/4) of providers: self-efficacy, opportunity, and social support (Table 4; constructs have been grouped based on the Theoretical Domains Framework [52]).

None of the 4 digital providers linked all of their planned BCTs (listed in Table 2) to the proposed theoretical constructs, although provider B did link all constructs and intervention functions of the Capability, Opportunity, Motivation–Behavior model (which their intervention was solely based on) to some planned BCTs, which was detailed in their intervention design documents (see item 10 in Table 3). All providers linked at least some of their planned BCTs to theoretical constructs (see item 8a in Table 3). For example, provider A described modeling, goal setting, and problem solving to drive self-efficacy, and provider C described the techniques of verbal persuasion, reviewing goals, and social support to target self-efficacy.

**Table 3:** Use of theory in digital providers’ intervention designs^a^.

Use of theory (item^b^)	A	B	C	D
Theory mentioned (1a)	✓	✓	✓	✓
Construct mentioned (1b)^c^	✓	✓	✓	✓
Target construct mentioned as a predictor of behavior (2)	✓	✓	✓	✓
Intervention based on a single theory (3)		✓		✓
Theory or predictors used to select recipients for the intervention (4)				
Theory or predictors used to select or develop intervention techniques (5)	✓	✓	✓	✓
Theory or predictors used to tailor intervention techniques to recipients (6)	✓	✓	✓	
All intervention techniques are explicitly linked to at least one theory-relevant construct or predictor (7a)				
All intervention techniques are explicitly linked to an overall *theory* or *model* but not a specific construct (7b)^c^				
At least one but not all of the intervention techniques are explicitly linked to at least one theory-relevant construct or predictor (8a)	✓	✓	✓	✓
At least one but not all of the intervention techniques are explicitly linked to an overall *theory* or *model* but not a specific construct (8b)^c^				
The group of techniques is linked to a group of constructs or predictors (9a)	✓		✓	
The group of techniques is linked to an overall *theory* or *model* but not a specific construct (9b)^c^				
All theory-relevant constructs or predictors are explicitly linked to at least one intervention technique (10)		✓		
At least one but not all of the theory-relevant constructs or predictors are explicitly linked to at least one intervention technique (11)	✓^d^		✓	✓
Theory-relevant constructs or predictors are measured (12)				
Quality of measures (13)				

^a^The data in this table combine the results from the design documentation supplied by providers and interviews with program developers involved in the design of the digital diabetes prevention program.

^b^Denotes items of the Theory Coding Scheme [25]; items 14 to 19 of the Theory Coding Scheme relate to postintervention rather than protocol assessment and are therefore not included in this analysis.

^c^Additional items that the authors added to the Theory Coding Scheme for this analysis (Multimedia Appendix 2).

^d^Provider linked all constructs and intervention functions of the Capability Opportunity Motivation–Behavior model [37] to behavior change techniques but did not link all other listed theoretical constructs to behavior change techniques.

**Table 4:** Theories, approaches, and constructs mentioned in each digital providers’ diabetes prevention program designs^a^.

Models, approaches, constructs, and concepts		A	B	C	D
**Model of behavior (change) mentioned**					
	Behavior change wheel [41]			✓	✓
	Biopsychosocial model [42]		✓		
	Cognitive behavioral theory [43]	✓			
	COM-B^b^ model [37]	✓	✓		✓
	Functional Learning Theory [44]	✓			
	GROW^c^ model [45]			✓	
	Health Action Process Approach [46]	✓			
	Self-Determination Theory [40]			✓	
	Social Cognitive Theory [39]	✓		✓	
	Social Comparison Theory [47]	✓			
	Transtheoretical model [39]	✓		✓	
**Approaches to behavior change mentioned**					
	Acceptance and commitment therapy [48]	✓		✓	
	Cognitive behavioral therapy [49]	✓	✓	✓	
	Motivational interviewing [50]	✓	✓	✓	
	Positive psychology [51]			✓	
**Constructs^d^ mentioned**					
	Autonomy			✓	
	Behavior regulation (internal), self-control, and self-management	✓			✓
	Beliefs about consequences, benefits and risks, personal beliefs, and risk perceptions	✓		✓	✓
	Capability, cognitive and interpersonal skills, competence, and physical skills	✓	✓	✓	✓
	Goals	✓	✓	✓	✓
	Intentions				✓
	Knowledge	✓			✓
	Motivation	✓	✓	✓	✓
	Intrinsic and extrinsic motivation			✓	
	Opportunity	✓	✓		✓
	Optimism				✓
	Reinforcement	✓			✓
	Self-efficacy, beliefs about capabilities, and observational learning	✓		✓	✓
	Social context, environmental context, and environmental influences			✓	✓
	Social support and relatedness	✓		✓	✓
**Behavior change concepts mentioned**					
	Accountability				✓
	Approach-avoidance language			✓	
	Behavioral influences			✓	
	Committed action			✓	
	Connectedness			✓	
	Lapses and relapses	✓		✓	

^a^The data in this table combine the results from the design documentation supplied by providers and interviews with program developers involved in the design of the digital diabetes prevention program.

^b^COM-B: Capability, Opportunity, Motivation–Behavior.

^c^GROW: Goal, Reality, Options, and Way forward.

^d^Constructs were grouped together based on the Theoretical Domains Framework [52].

## Discussion

### Principal Findings

The digital providers of the NHS-DDPP demonstrated good fidelity to the BCT content of the NHS-DDPP design. In particular, self-regulatory BCTs, which may be most effective in changing health behaviors [13], were included in the designs of all 4 digital providers. However, providers appeared to have inconsistent use of theory to inform their intervention designs, and none had developed a logic model or provided an explicit description of how their digital programs were expected to achieve the desired health outcomes. Without a clear underpinning theory and identification of the mechanisms underlying particular BCTs, the justification for the inclusion of specific BCT content in digital providers’ programs is not clear.

### Comparison With Prior Work

Overall, digital providers planned to deliver an average of 85% of the BCT content specified in the NHS-DPP program specification. Thus, the fidelity of design in the digital offering of the NHS-DPP is, in principle, higher than that previously reported for the face-to-face offering, where the fidelity of design was 74% [17]. The planned self-regulatory BCT content is particularly encouraging, given that these BCTs are backed by evidence to suggest that such techniques are most effective in changing health behaviors such as diet and physical activity [13]. However, the inconsistent use of theory in digital programs is similar to the evaluation of face-to-face NHS-DPP, which also highlighted a lack of mapping of BCTs to theoretical constructs and a lack of a logic model produced by any of the providers [27]. Both findings could be explained by the fact that providers’ digital programs were adapted from pre-existing digital interventions already implemented before the commissioning of the NHS-DDPP service. This may be a particular problem for digital interventions.

### Strengths and Limitations

This study provides a detailed analysis of the planned behavior change content of each of the 4 digital providers commissioned to deliver the NHS-DDPP using standardized coding frameworks [14,25], which demonstrated high κ agreement between coders. All framework response documentation obtained from each digital provider was submitted to the commissioners during service procurement; thus, they should represent an accurate description of digital providers’ intervention designs. In addition, the research team requested any other relevant documentation from each digital provider that contained information about the planned behavior change content of the NHS-DDPP. Providers were given multiple opportunities to share all relevant documentation with the research team to support the current analysis.

However, one of the providers was reluctant to share all documentation detailing the theoretical underpinning because of the commercial sensitivity of these documents. Researchers attempted to obtain as much information as possible about the theoretical foundations of providers’ programs via interviews, especially when documentation could not be shared with the research team. The interviews were able to supplement the document review and were used to fill any gaps in the information that was not present in the documentation. Despite some reluctance to share documentation with the research team from one of the digital providers, analyses from this provider were similar to those of the other 3 providers, who shared all documentation. Furthermore, all digital providers confirmed that they had not developed a logic model to explicitly map how their digital programs would achieve the desired program outcomes, nor was there an alternative presentation of how their interventions were expected to work (eg, via free text).

It should also be noted that participants who were interviewed for this study were not always necessarily directly involved in the development of the BCT content and the theoretical underpinning of the programs. Some people involved in the early stages of design and development had since moved on to other roles. Nonetheless, the research team tried to identify ≥1 relevant individual from each digital provider and aimed to interview professionals from different backgrounds to gather a range of views and provide a comprehensive understanding of the processes involved in the design and development of each NHS-DDPP intervention.

### Future Directions

Digital interventions have the potential to be delivered with higher fidelity than face-to-face interventions [7] as they do not rely solely on facilitator-centered delivery; instead, the BCT content can be standardized in its delivery via the functions of an app or website. Therefore, the current fidelity of design results is encouraging, as drift in delivery fidelity is less likely, thus increasing the chances of the program being effective if the BCT content is proven to be engaged with effectively.

Despite the encouraging results with regard to the planned BCTs in digital providers’ program designs, there is inconsistent use of theory from providers to justify why specific BCTs have been included and how they will produce the desired behavior changes (ie, the theoretical constructs they are targeting). Given that a clear underpinning theory supports the translation of BCTs in intervention designs to intervention delivery [24-26], the absence of a clear logic model or an explicit explanation of underpinning theory describing the constructs to be targeted by specific BCTs is potentially problematic.

One of the ways in which providers could usefully describe the underpinning theory is via a logic model, which provides a concise and clear way of visually presenting interventions informed by multiple theories. However, there is no clear guidance on whether it should be the role of the providers to produce a logic model or whether it would be more appropriate for commissioners of large-scale programs to produce an initial logic model to guide providers from the outset [27]. However, the requirement of a logic model during the commissioning process of large-scale programs could ensure a clear rationale for the BCTs included in intervention designs, which may result in fewer issues in delivery and receipt fidelity in program implementation [27]. It should be noted that the inclusion of a logic model is a recommendation that has emerged since the evaluation of the NHS-DPP commenced and following the previous round of service procurement. Commissioners of the program are now increasingly focusing on the important role of logic models in the upcoming round of service procurement for the NHS-DPP.

In addition, a further 68% of the planned BCT content in the digital service was not specified in the program specification, a finding similar to the face-to-face offering of the NHS-DPP [17]. It is possible that interventions containing more techniques to help people change their diet and physical activity behaviors may be more effective [53,54]. However, without a logic model or explicit description of the underpinning theory justifying the inclusion of additional BCT content, programs cannot be tested and evaluated effectively to establish the techniques that work in digital behavior change interventions and why.

This research provides an example of a design fidelity assessment of a large-scale digital intervention. Using the present findings as a baseline, future research will assess fidelity of the delivery of the digital service and service user engagement with the program. The current analysis found that some digital providers still plan to rely on health coaches to deliver aspects of the program to service users (eg, support via telephone); thus, the nature of this delivery will depend on the skills of the health coach in a similar way as that delivered by facilitators of face-to-face sessions. This may help to increase program engagement [55] but may also pose a risk to delivery fidelity because of variations in the background or training of health coaches. This study will be critical to assess whether the plans digital providers state they will put in place (with high fidelity) translate into program content that is actually delivered and understood as intended. Ongoing qualitative work examines how the behavior change content of the program is understood by service users, an underresearched aspect of fidelity [56]. Further analysis of the interview data with program developers from this study is also planned, with the aim of qualitatively capturing the nuances of intervention development and how each of the digital providers’ programs has evolved over time.

### Conclusions

When designing their programs, the digital providers of the NHS-DDPP demonstrated good fidelity to the BCT content of the program specification. Thus, if the NHS-DDPP is delivered with high fidelity and engaged with by service users, it should increase the effectiveness of the program in achieving a reduction in the incidence of T2DM. However, the lack of theory described in digital providers’ intervention designs is potentially problematic if providers are not explicit in how their programs will work to achieve behavior change, given that a clear underpinning theory supports the translation of BCT content in intervention design to intervention delivery.

## Appendix

Multimedia Appendix 1 National Institute of Health and Care Excellence NG183 guidance (2020)—Behavior Change: Digital interventions.

Multimedia Appendix 2 Coding procedures.

Multimedia Appendix 3 Cohen κ values.

Multimedia Appendix 4 Behavior change technique definitions.

## Abbreviations

BCT: behavior change technique
BCTTv1: Behavior Change Technique Taxonomy version 1
DIPLOMA: Diabetes Prevention–Long Term Multimethod Assessment
IRR: interrater reliability
NHS: National Health Service
NHS-DDPP: National Health Service Digital Diabetes Prevention Program
NHS-DPP: National Health Service Diabetes Prevention Program
NICE: National Institute for Health and Care Excellence
T2DM: type 2 diabetes mellitus
TCS: Theory Coding Scheme

## Data Availability

The intervention design documentation from digital providers and audio recordings of interviews analyzed in this study are not publicly available because of confidentiality agreements with the provider organizations, as some information is commercially sensitive. Some data sets are available from the corresponding author on reasonable request, although the authors will require explicit permission from the relevant provider organizations.
