# Supplementary material for: Fidelity to Program Specification of the National Health Service Digital Diabetes Prevention Program Behavior Change Technique Content and Underpinning Theory: Document Analysis
Source: J Med Internet Res. 2022 Apr 27;24(4):e34253. doi: 10.2196/34253 (PMC9096650; doi:10.2196/34253)
Supplement: Multimedia Appendix 1 [file jmir_v24i4e34253_app1.docx]

**Appendix A: NICE NG183 Guidance (2020) – ‘Behaviour Change: Digital interventions’**

**Table A1. Recommendations in NICE NG183 Guideline (2020) relevant to the NHS-DDPP**

| **NICE NG183 Recommendation** | **Comments** |
| --- | --- |
| **Behaviour Change Content** | |
| 1.1.3: When designing digital and mobile health interventions, use evidence-based behaviour change techniques that help people start and maintain changes. These include: **goals and planning, feedback and monitoring, and social support.** | These BCTs are already present in the NICE PH38 guideline, which also emphasises self-regulatory BCTs. |
| 1.4.2: Advise people to use digital and mobile health interventions that include **self-monitoring**, such as recording by activity trackers, or food or physical activity diaries. This can help the person to **review their own progress towards their diet or physical activity goals**. |  |
| **Service Delivery Parameters** | |
| 1.1.4: Consider designing interventions that allow the user to tailor goals to their own needs. | These recommendations are relevant to the service delivery parameters of digital interventions, but not the specific behaviour change *content* (i.e. BCTs and underpinning theory) which are described in the current study. |
| 1.1.7: Make information available about:  - How users can check and set preferences for how their personal information and data may be used  - When the intervention is likely to use mobile data, and how much mobile data it is likely to use  - Any additional costs  - Terms and conditions. |  |
| 1.1.8: When developing digital and mobile health interventions, involve a wide range of stakeholders, including potential users, as early as possible and throughout development to:  - Develop and review the content, structure, interface and flow of the intervention.  - Identify the best digital platforms for the target population.  - Identify and address any aspects of the intervention that may unintentionally increase inequity and digital exclusion.  - Discuss and ensure that users understand who the intervention is for, which behaviour it is trying to change, its aims, any possible harms, the time needed to establish behaviour change and how frequently users are likely to interact with the intervention. |  |
| 1.1.9: Use feedback from testing and after releasing the intervention to continually improve the intervention. |  |

Reference: National Institute for Health and Care Excellence (NICE). NG183, Behaviour change: Digital and mobile health interventions. London: National Institute for Health and Care Excellence. 2020. <https://www.nice.org.uk/guidance/ng183>
