# Supplementary material for: Fidelity to Program Specification of the National Health Service Digital Diabetes Prevention Program Behavior Change Technique Content and Underpinning Theory: Document Analysis
Source: J Med Internet Res. 2022 Apr 27;24(4):e34253. doi: 10.2196/34253 (PMC9096650; doi:10.2196/34253)
Supplement: Multimedia Appendix 2 [file jmir_v24i4e34253_app2.docx]

**Appendix B: Coding Procedures**

Data were extracted from the relevant documentation and interview transcripts using the BCTTv1 and TCS by one researcher (REH). The framework response bids from providers’ intervention design documentation were double coded for BCTs and underpinning theory by a second researcher (LMM). Authors used a new author-developed data extraction sheet for each separate document and each individual interview for each provider.

Behaviour Change Technique Coding Procedures

BCTs were coded using an author-developed data extraction sheet. Researchers underwent training in the use of the BCTTv1 (<https://www.bct-taxonomy.com/>) and a set of coding rules were developed through team discussions. Behaviour change techniques (BCTs) were coded using an author-developed table, which included the following columns:

- BCT label
- Confidence of presence
- Information about behavioural targets (e.g. diet, physical activity)
- Mode of delivery (e.g. visual, auditory, text)
- Interactive vs. passive delivery of BCT (i.e. whether the planned BCT required action from the patients)
- Whether the planned BCT was optional or compulsory (e.g. an optional interactive activity)
- Location in the document/interview transcript (defined by page number and page quarter)
- Summarised evidence of the planned BCT

BCT coding rules stated that new BCTs would be coded on the commencement of a new activity or if a different health behaviour (e.g. diet, physical activity) was targeted. The level of target behaviour was also documented when coding the BCT ‘information about health consequences’ (e.g., levels of the target behaviour ‘diet’ included information about carbohydrates, fats, sugar, etc.) as the authors felt these were distinct pieces of information targeting distinct behaviours.

BCTs present in both the full programme specification and digital providers’ intervention designs were documented as indicating fidelity to the programme specification. BCTs stated in the full programme specification that were not present in the digital intervention designs, and additional BCTs identified in the design documentation and interview transcripts which were not otherwise specified, were documented as indicating non-fidelity to the programme specification.

Data Extraction of Underpinning Theory

A data extraction sheet was developed using Michie and Prestwich’s (2010) TCS. Authors removed items 14-19 of this coding scheme as these items related to post-intervention rather than protocol assessment. The following items were also added to the TCS to ensure all relevant theoretical content was captured:

- Item 1b: ‘A construct was mentioned’, this was added to capture a construct or predictor that was mentioned but not linked to behaviour.
- Item 7b: ‘All intervention techniques are explicitly linked to an overall theory/model but not a specific construct’.
- Item 8b: ‘At least one, but not all, of the intervention techniques are explicitly linked to an overall theory/model but not a specific construct’.
- Item 9b: ‘Group of techniques are linked to an overall theory/model but not a specific construct’.
