# Supplementary material for: Fidelity to Program Specification of the National Health Service Digital Diabetes Prevention Program Behavior Change Technique Content and Underpinning Theory: Document Analysis
Source: J Med Internet Res. 2022 Apr 27;24(4):e34253. doi: 10.2196/34253 (PMC9096650; doi:10.2196/34253)
Supplement: Multimedia Appendix 3 [file jmir_v24i4e34253_app3.docx]

**Appendix C: Kappa Values**

**Table A2. Kappa Values for NHS-DDPP Intervention Design Documents**

| **Source document** | **Kappa value** |
| --- | --- |
| NHS Service Specification  NICE PH38 Guideline | 0.84 0.87 |
| **Provider A**   BCT coding   Theory coding scheme   Theory mentioned   Constructs mentioned | 0.82  0.92  1.00  0.90 |
| **Provider B**   BCT coding   Theory coding scheme   Theory mentioned   Constructs mentioned | 0.89  0.96  1.00  1.00 |
| **Provider C**   BCT coding   Theory coding scheme   Theory mentioned   Constructs mentioned | 0.66  0.92  1.00  1.00 |
| **Provider D**   BCT coding   Theory coding scheme   Theory mentioned   Constructs mentioned | 0.93  0.80  1.00  0.96 |
