# Supplementary material for: Fidelity to Program Specification of the National Health Service Digital Diabetes Prevention Program Behavior Change Technique Content and Underpinning Theory: Document Analysis
Source: J Med Internet Res. 2022 Apr 27;24(4):e34253. doi: 10.2196/34253 (PMC9096650; doi:10.2196/34253)
Supplement: Multimedia Appendix 4 [file jmir_v24i4e34253_app4.docx]

**Appendix D: Behaviour Change Technique Definitions**

**Table A3. Behaviour Change Technique Definitions**

| **Behaviour Change Technique** | **Definition** |
| --- | --- |
| Goal setting for health behaviours [1.1] | Set or agree on a goal defined in terms of the behaviour to be achieved. |
| Problem solving [1.2] | Prompt the person to analyse factors influencing the behaviour and generate or select strategies that include overcoming barriers or increasing facilitators. |
| Goal setting for health outcomes [1.3] | Set or agree on a goal defined in terms of a positive outcome of wanted behaviour. |
| Action planning [1.4] | Prompt detailed planning of the performance of the behaviour (must include at least one of context, frequency, duration and intensity). |
| Reviewing behaviour goals [1.5] | Review behaviour goal(s) jointly with the person and consider modifying goal(s) or behaviour change strategy in light of achievement. |
| Discrepancy between current behaviour and goal [1.6] | Draw attention to discrepancies between a person’s current behaviour (in terms of the *form, frequency, duration, or intensity* of that behaviour) and the person’s previously set outcome goals, behavioural goals or action plans. |
| Reviewing outcome goals [1.7] | Review outcome goal(s) jointly with the person and consider modifying goal(s) in light of achievement. |
| Behavioural contract [1.8] | Create a written specification of the behaviour to be performed, agreed on by the person, and witnessed by another |
| Commitment [1.9] | Ask the person to affirm or reaffirm statements indicating commitment to change the behaviour. |
| Giving feedback on behaviour [2.2] | Monitor and provide informative or evaluative feedback on performance of the behaviour. |
| Self-monitoring of behaviour [2.3] | Establish a method for the person to monitor and record their behaviour(s) as part of a behaviour change strategy. |
| Self-monitoring of outcomes of behaviour [2.4] | Establish a method for the person to monitor and record the outcome(s) their behaviour as part of a behaviour change strategy. |
| Monitoring outcome of behaviour by others without feedback [2.5] | Observe or record outcomes of behaviour with the person’s knowledge as part of a behaviour change strategy. |
| Biofeedback [2.6] | Provide feedback about the body *(e.g. physiological or biochemical state)* using an external monitoring device as part of a behaviour change strategy. |
| Giving feedback on outcomes of behaviour [2.7] | Monitor and provide feedback on the outcome of performance of the behaviour. |
| Unspecified social support [3.1] | Advise on, arrange or provide social support or non-contingent praise or reward for performance of the behaviour. |
| Practical social support [3.2] | Advise on, arrange or provide practical help for performance of the behaviour. |
| Emotional social support [3.3] | Advise on, arrange or provide emotional social support for performance of the behaviour. |
| Instruction on how to perform a behaviour [4.1] | Advise or agree on how to perform the behaviour (includes ‘Skills training’). |
| Information about antecedents [4.2] | Provide information about antecedents (*e.g. social and environmental situations and events, emotions, cognitions)* that reliably predict performance of the behaviour. |
| Behavioural experiments [4.4] | Advise on how to identify and test hypotheses about the behaviour, its causes and consequences, by collecting and interpreting data. |
| Information about health consequences [5.1] | Provide information about health consequences of performing the behaviour. |
| Salience of consequences [5.2] | Use methods specifically designed to emphasise the consequences of performing the behaviour with the aim of making them more memorable (goes beyond informing about consequences). |
| Salience of behaviours ^a^ | Use methods specifically designed to emphasise the behaviour when linking the behaviour to the consequence with the aim of making the consequence more memorable. |
| Giving information about social and environmental consequences [5.3] | Provide information (e.g. written, verbal, visual) about social and environmental consequences of performing the behaviour. |
| Monitoring of emotional consequences [5.4] | Prompt assessment of feelings after attempts at performing the behaviour. |
| Anticipated regret [5.5] | Induce or raise awareness of expectations of future regret about performance of the unwanted behaviour. |
| Giving information about emotional consequences [5.6] | Provide information (e.g. written, verbal, visual) about emotional consequences of performing the behaviour. |
| Giving a demonstration of the behaviour [6.1] | Provide an observable sample of the performance of the behaviour, directly in person or indirectly e.g. via film, pictures, for the person to aspire to or imitate. |
| Social comparison [6.2] | Draw attention to others’ performance to allow comparison with the person’s own performance. |
| Prompts/cues [7.1] | Introduce or define environmental or social stimulus with the purpose of prompting or cueing the behaviour. |
| Remove access to the reward [7.4] | Advise or arrange for the person to be separated from situations in which unwanted behaviour can be rewarded in order to reduce the behaviour. |
| Remove aversive stimulus [7.5] | Advise or arrange for the removal of an aversive stimulus to facilitate behaviour change. |
| Behavioural practice [8.1] | Prompt practice or rehearsal of the performance of the behaviour in order to increase habit or skill. |
| Behaviour substitution [8.2] | Prompt the substitution of the unwanted behaviour with a wanted or neutral behaviour. |
| Habit formation [8.3] | Prompt rehearsal and repetition of the behaviour in the same context repeatedly so that the context elicits the behaviour. |
| Habit reversal [8.4] | Prompt rehearsal and repetition of an alternative behaviour to replace an unwanted habitual behaviour. |
| Overcorrection [8.5] | Ask to repeat the wanted behaviour in an exaggerated way following an unwanted behaviour. |
| Graded tasks [8.7] | Set easy-to-perform tasks, making them increasingly difficult, but achievable, until behaviour is performed. |
| Credible source [9.1] | Present verbal or visual communication from a credible source in favour of or against the behaviour. |
| Pros and cons [9.2] | Advise the person to identify and compare reasons for wanting (pros) and not wanting to (cons) change the behaviour. |
| Comparative imagining of future outcomes [9.3] | Prompt or advise the imagining and comparing of future outcomes of changed versus unchanged behaviour. |
| Material incentive (behaviour) [10.1] | Inform that money, vouchers or other valued objects will be delivered if and only if there has been effort and/or progress in performing the behaviour. |
| Material reward (behaviour) [10.2] | Arrange for the delivery of money, vouchers or other valued objects if and only if there has been effort and/or progress in performing the behaviour. |
| Social reward [10.4] | Arrange verbal or non-verbal reward if and only if there has been effort and/or progress in performing the behaviour. |
| Social incentive [10.5] | Inform that a verbal or non-verbal reward will be delivered if and only if there has been effort and/or progress in performing the behaviour. |
| Non-specific incentive [10.6] | Inform that a reward will be delivered if and only if there has been effort and/or progress in performing the behaviour. |
| Self-incentive [10.7] | Plan to reward self in future if and only if there has been effort and/or progress in performing the behaviour. |
| Self-reward [10.9] | Prompt self-praise or self-reward if and only if there has been effort and/or progress in performing the behaviour. |
| Reward (outcome) [10.10] | Arrange for the delivery of a reward if and only if there has been effort and/or progress in achieving the behavioural outcome. |
| Pharmacological support [11.1] | Provide, or encourage the use of or adherence to, drugs to facilitate behaviour change. |
| Reduce negative emotions [11.2] | Advise on ways of reducing negative emotions to facilitate performance of the behaviour. |
| Increase positive emotions ^b^ | Advise on ways of increasing positive emotions to facilitate performance of the behaviour. |
| Restructuring the physical environment [12.1] | Change, or advise to change the physical environment in order to facilitate performance of the wanted behaviour or create barriers to the unwanted behaviour. |
| Restructuring the social environment [12.2] | Change, or advise to change the social environment in order to facilitate performance of the wanted behaviour or create barriers to the unwanted behaviour. |
| Avoiding/reducing exposure to cues for the behaviour [12.3] | Advise on how to avoid exposure to specific social and contextual/physical cues for the behaviour, including changing daily or weekly routines. |
| Distraction [12.4] | Advise or arrange to use an alternative focus for attention to avoid triggers for unwanted behaviour. |
| Adding objects to the environment [12.5] | Add objects to the environment in order to facilitate performance of the behaviour. |
| Body changes [12.6] | Alter body structure, functioning or support directly to facilitate behaviour change. |
| Identification of self as role model [13.1] | Inform that one's own behaviour may be an example to others. |
| Framing/reframing [13.2] | Suggest the deliberate adoption of a perspective or new perspective on behaviour (e.g. its purpose) in order to change cognitions or emotions about performing the behaviour. |
| Reward alternative behaviour [14.8] | Arrange reward for performance of an alternative to the unwanted behaviour. |
| Verbal persuasion about capability [15.1] | Tell the person that they can successfully perform the wanted behaviour, arguing against self-doubts and asserting that they can and will succeed. |
| Mental rehearsal of successful performance [15.2] | Advise to practise imagining performing the behaviour successfully in relevant contexts. |
| Focus on past success [15.3] | Advise to think about or list previous successes in performing the behaviour (or parts of it). |
| Self-talk [15.4] | Prompt positive self-talk (aloud or silently) before and during the behaviour. |
| Vicarious consequences [16.3] | Prompt observation of the consequences (including rewards and punishments) for others when they perform the behaviour. |

*Note: Definitions are summarised from BCTTv1. Numbers in square brackets are corresponding number in BCTTv1.*

*^a^ Salience of behaviours was not listed in the BCTTv1, but has been identified as a new behaviour change technique by the authors of this paper.*

*^b^ Increase positive emotions is not listed in the BCTTv1, but was noted by the authors for inclusion in the next version of the taxonomy.*
